# Natural killer cell immunotherapy to target stem-like tumor cells

**DOI:** 10.1186/s40425-016-0124-2

**Published:** 2016-04-19

**Authors:** Steven K. Grossenbacher, Robert J. Canter, William J. Murphy

**Affiliations:** Department of Dermatology, University of California Davis School of Medicine, Sacramento, CA 95817 USA; Division of Surgical Oncology, Department of Surgery, University of California Davis School of Medicine, Sacramento, CA 95817 USA; Division of Hematology and Oncology, Department of Internal Medicine, University of California Davis Medical Center, Sacramento, CA 95817 USA

## Abstract

Advances in cancer immunotherapy are leading to its increasing and successful application for the treatment of solid–tissue cancers. Despite the recent advances there are still significant barriers, in particular, evidence for significant tumor heterogeneity, both genetic and epigenetic that limit long-term efficacy. Subpopulations of “stem-like” tumor cells have been identified in nearly all human malignancies based on both morphologic and functional criteria. Also called cancer stem cells or CSCs, these quiescent cells display enhanced tumorigenic potential and are capable of repopulating tumors in the wake of traditional cytoreductive therapies. These CSCs may be best targeted via immunotherapy. Our lab has identified activated natural killer (NK) cell-based therapy as an effective method to target CSCs particularly after radiation therapy for solid tumors. Using a variety of in vitro and in vivo methods, including the utilization of primary tumor tissue and patient-derived xenografts, we observed that autologous and allogeneic NK cells possess the ability to preferentially kill stem-like cells or CSCs from freshly isolated patient samples representing a broad spectrum of tumor types, including pancreatic cancers, breast cancers, and sarcomas. The results indicated that CSCs express stress ligand molecules capable of being targeted by NKG2D on NK cells and that prior radiation therapy can both deplete the cycling non-CSCs bulk tumor population and upregulate these stress ligands on the CSC making this an effective combination approach.

## Background

Recent strategies to activate and engage the immune system have led to significant breakthroughs in cancer therapy. The recent success of checkpoint blockade therapy, targeting the immune-inhibitory proteins PD-1 and/or CTLA-4, has initiated a paradigm shift in the clinical and preclinical investigation and use of immunotherapy for solid tumors. However, despite the impressive clinical efficacy of the most recent iterations of these therapies, there are still significant issues that remain. Stem-like tumor cells, also known as cancer stem cells (CSCs), are present in numerous hematological and solid malignancies [1, 2]. However, there is considerable debate regarding how to properly characterize these cells, whether they exist as a discrete subpopulation or are a plastic phenotype under epigenetic control, and a variety of correlative marker systems have been suggested for various tissue types. Classically, a very small fraction of CSCs possesses the ability to form aggressively growing, heterogeneous, tumors in immunodeficient mice or in long-term in vitro outgrowth assays. Depending on the tissue of origin, the cell surface markers CD133, CD24/CD44, as well as high expression of aldehyde dehydrogenase (ALDH) delineates these cells from their more differentiated and rapidly dividing progeny. Additionally, the use of extensively cultured commercial cell lines to study these cells does not accurately represent the heterogeneity found within primary human tumors, where the epigenetic influence of certain microenvironmental factors, such as hypoxia and/or inflammation exists [3]. To combat this discrepancy, conclusions drawn from tumor cell lines are being increasingly supported by data using human primary tumor tissues. We, and others, observed that despite reduction in overall tumor size with conventional therapies, such as radiotherapy, chemotherapy, and small molecule inhibitors, residual stem-like tumor cells remain, which have the ability to repopulate the tumor mass and seed metastases [4–7]. An immune approach to target this population of cells is likely to fill an unmet need, since unlike chemotherapy, targeted small molecule therapy, and radiotherapy; immune cells do not need tumor cell targets to be actively cycling in order to mediate their effects. We postulated that the use of immunotherapy after such regimens are applied may allow for greater CSC targeting, particularly when used after cytoreductive therapies.

## Main text

Our laboratory has explored the use of natural killer (NK) cells in the recognition and targeting of CSCs in several human solid cancers. NK cells are terminally differentiated innate lymphoid cells with the ability to spontaneously kill virally-infected or malignant cells without prior immunization in an MHC-unrestricted manner. As an adoptive immunotherapy, NK cells have primarily been used against hematologic cancers as results with solid tissue tumors have been disappointing [8–10]. The goal of our studies was to determine if NK cells would selectively identify and lyse stem-like tumor cells and to understand how this potential recognition occurs. NK cells have been long known for their unique ability to reject allogeneic hematopoietic stem cells, but not solid tissue allografts. Previous data from our laboratory demonstrated that NK cells reject embryonic stem cells in an NKG2D-dependent manner and thus, we sought to apply this same rationale to evaluate their potential to target CSCs [11]. We have demonstrated that certain small molecule drugs and radiation therapy enrich for populations of tumor cells that harbor stem-like qualities and may possibly sensitize these CSCs to NK cell attack [4, 6].

In our initial evaluation, we screened several human breast, brain, pancreas, and sarcoma tumor cell lines for their susceptibility to cytokine-activated NK cell-mediated cytotoxicity [12]. Using flow cytometry, we observed that activated allogeneic NK cells significantly killed stem-like tumor cells expressing high amounts of the CSC-associated proteins ALDH, CD133, CD24, or CD44. Importantly, our cell line data was mirrored when we evaluated a panel of primary tumor cells derived from freshly excised patient samples. These primary tissue samples were tested within 48 h after surgical resection and were comprised of unsorted cells from multiple subtypes of sarcoma as well as multiple pancreatic adenocarcinoma samples. It is important to note that while NK cells demonstrated the capacity to kill both the CSC and non-CSC populations, the greatest decreases in the overall frequency of cells before and after NK killing occurred within the CSCs. In order to address the possibility of patient/donor MHC mismatching contributing to the CSC killing we observed, we also compared autologous versus allogeneic NK cell activity. We observed similar efficacy in the ability of activated NK cells to lyse stem-like targets in vitro although since these cells were highly cytokine-activated first, it does not rule out potential differences with lower activation. Other groups have noted that certain populations of stem-like tumor cells express various ligands for NK cell activating receptors [13]. We observed that CSCs from multiple solid tumors expressed ligands for the NK cell activating receptor NKG2D, and that blocking NKG2D using Fc-chimeric proteins abrogated the NK killing in vitro. Lastly, in order to evaluate the translational potential of the effects we observed in vitro, we implanted NSG mice with human tumor xenografts and evaluated the effects of adoptively transferred NK cells to eliminate stem-like cells in vivo. Paralleling our in vitro results, we observed that activated allogeneic NK cells significantly reduced the frequencies of CSCs, reduced the colony forming capacity of treated tumor cells ex vivo, and delayed the growth of orthotopic pancreatic xenograft tumors. Importantly, we have also recently observed that combining NK cell therapy with prior local tumor irradiation markedly enhances these effects, indicating that for optimal efficacy that elimination of the non-CSC populations needs to occur [4].

## Conclusions

Immunotherapies appear to have promise for the goal of attacking the heterogeneity of cancers based on the ability of immune cells to indiscriminately kill tumor cells in different phases of the cell cycle, unlike traditional chemotherapy and radiotherapy. It is apparent that heterogeneity within cancer exists with subpopulations of stem-like cells or CSCs exhibiting resistance to conventional cytoreductive therapies possibly due to quiescence as well as other pathways. Immunotherapy with T cells often results in antigen-loss variants within the tumor. We have demonstrated that activated NK cells may be uniquely capable of targeting CSCs of solid tissue cancers via stress ligand recognition. Future studies will need to evaluate the immunological impact of NK cell killing of CSCs using immunocompetent models as this may aid in augmenting later T cell responses. It will also be critical to optimize NK cell immunotherapy with regard to sustained in vivo effects as the highly activated NK cells are dependent on cytokines for continued function and survival. Greater targeting of CSCs by the NK cells, possibly through monoclonal antibodies may also increase efficacy. It is likely that NK cells need to be applied using a combination therapy approach as reduction of non-CSCs, which comprise the bulk of the tumor, needs to occur. As our understanding of the nature of the stem-like or CSC subpopulations continues to evolve, so too will our ability to apply immunotherapy more effectively.

## Abbreviations

ALDH: Aldehyde dehydrogenase
CD: Cluster of differentiation
CSC: Cancer stem cell
Fc: Fragment, crystallizable
MHC: Major histocompatibility complex
NK: Natural killer
NKG2D: Natural killer group 2, member D
